# Medial prefrontal glutamate response to acute stress is associated with social subordination in female rhesus macaques

**DOI:** 10.1038/s41398-025-03334-2

**Published:** 2025-03-29

**Authors:** Michael T. Treadway, Samantha A. Betters, Jessica A. Cooper, Chun-Xia Li, Xiaodong Zhang, Vasiliki Michopoulos

**Affiliations:** 1 Department of Psychology, Emory University, Atlanta, GA USA; 2 Department of Psychiatry and Behavioral Sciences, Emory School of Medicine, Atlanta, GA USA; 3 Emory National Primate Research Center, Atlanta, GA USA

**Keywords:** Physiology, Diagnostic markers, Molecular neuroscience

## Abstract

Chronic psychosocial stress is associated with increased risk of psychiatric disorders. Magnetic resonance spectroscopy (MRS) in humans has been used to show that glutamate levels in medial prefrontal cortex (mPFC) following acute stress exposure adapt to recent chronic stress levels. Here, we sought to determine the presence of this glutamate stress response adaptation in rhesus macaques, whose societies are maintained by dominance relationships that are enforced by agonistic interactions and result in chronic stress phenotypes seen in humans. We tested the hypothesis that change in mPFC glutamate after an acute stressor would be moderated by behavioral factors related to social subordination in a manner similar to that previously observed in humans. Seventeen adult female rhesus monkeys (*Macaca mulatta*, 13–23 yrs.) were observed over ten weeks to collect behavioral data and then received two MRS scans. The first scan occurred after acute stress manipulation involving relocation and isolation. The second control scan occurred after acclimation to the new location. As expected, we found that a behavioral measure of social subordination predicted an adaptive glutamate response such that animals experiencing more submissive behavior asymmetry (a behavioral measure related to social subordination) exhibited an attenuated glutamate response to the acute stressor. These data establish the use of MRS to measure the adaptive glutamate stress in non-human primates and will help further our understanding of the neurobiology of stress adaptation.

## Introduction

The role of stress in the etiology of psychiatric disease has been well-established [1–6], yet the pathophysiology of stress-linked disease remains obscure. This is in part due to the enormous capacity for resilience and adaptation in the face of stress exposure that organisms display. Indeed, mild stress exposure is believed to stimulate protective or inoculating biological processes that mitigate consequences for subsequent stressor exposure [7–9]. To date, human magnetic resonance spectroscopy (MRS) neuroimaging methods have highlighted a potentially critical role for the glutamate system [10–14] in the pathophysiology of stress-related disorders, but the directionality of the result has been mixed across studies (see [15] for a review). Consequently, the precise role of glutamate in adaptive and maladaptive responses to stress remain unclear.

In rodent models, the medial prefrontal cortex (mPFC) has been repeatedly implicated in mediating adaptative and maladaptive responses to stress. Upon acute stressor onset, mPFC catecholamine and glutamate levels rise rapidly [16–18], and continued excitation of mPFC pyramidal neurons is facilitated by stress-induced increases in glucocorticoids [19, 20]. The effects of an acute stressor depend largely on chronicity [21]. For example, initial acute stress exposure increases extracellular glutamate and up-regulates surface expression of excitatory amino-acid receptors [16, 22, 23]. As stress exposure becomes more chronic, uncontrollable or unpredictable, however, glutamate release in response to subsequent acute stressors shows rapid habituation [16]. Similarly, animals previously exposed to chronic unpredictable stress demonstrate reduced potentiation of prefrontal glutamatergic signaling when faced with a subsequent stressor [24–26]. These results point to an allostatic regulatory process in the mPFC that adapts to repeated acute stress exposure via down-regulation of glutamatergic tone [27].

An unresolved question, however, is identifying the mechanisms that mediate adaptation to differing levels of stress exposure. To address this, our group recently sought to translate previous rodent work to humans using in vivo functional MR spectroscopy to evaluate mPFC glutamate change following exposure to an acute stressor [28]. Across two samples of healthy control participants recruited on the basis of a wide range of recent chronic stress (as assessed by the Perceived Stress Scale; “PSS” [29]) we found that mPFC glutamate levels increased in response to an acute laboratory stressor in individuals with low levels of chronic stress, but glutamate response to acute stress decreased as PSS scores increased, consistent with a putatively neuroprotective response observed in rodents. Interestingly, in unmedicated patients with major depressive disorder (MDD), chronic stress did not moderate glutamate response to an acute laboratory stressor, despite high PSS scores, suggesting that the adaptive glutamate response (“AGR”) that was present in multiple samples of healthy control participants was absent in MDD [28]. This altered response was in turn correlated with pessimistic expectations of future events in daily life. Taken together, our prior results suggest that the AGR is a potential mechanism supporting resilience to stress exposure.

A key limitation of this prior work in humans was its reliance on a relatively brief, retrospective self-report measure of perceived chronic stress. While the PSS is well-suited to capture perceptions of stress and capacity for coping in a general sense, it does not isolate particular types of stressors that may contribute to the AGR. Importantly, in highly socialized species such as humans and non-human primates (NHP) the links between stress and adverse behavioral outcomes are greatest for circumstances in which the stressor involves social subordination, rejection, or isolation [30–32]. Characterization of these social exposures in humans can be challenging, given the limited experimental control that researchers may exert over human exposure. In contrast, NHP models afford an excellent translational opportunity to assess the neurophysiological impacts of chronic psychosocial stressors like those that human experience.

One ethologically valid, translational NHP model of chronic psychosocial stress exposure, is social subordination in socially housed female rhesus monkeys. Socially housed female macaques, like human beings, live in stratified societies [33] wherein more lower ranking, subordinate females experience continuous harassment, typically induced by aggression from higher ranking group mates [34–37], and emit submissive behaviors towards higher ranking animals [33–36]. Social subordination in macaques has been linked to lower rates of affiliation with others (proximity and grooming) and greater rates of anxiety-like behavior [38, 39]. Importantly, social subordination in macaques is a potent psychosocial stressor that results in chronic diminished glucocorticoid negative feedback inhibition of the hypothalamic-pituitary-adrenal (HPA) axis [36, 40], increased peripheral pro-inflammatory gene expression [41, 42], and alterations in dopaminergic [43, 44], serotonergic [45, 46], and GABAergic systems [47] in prefrontal and limbic brain regions critical for emotion regulation.

In the current work, we sought to test for the presence of the AGR in a sample of dominant and subordinate socially housed female rhesus macaque monkeys (*n* = 17). Given prior work highlighting the relationship between social subordination and biological markers of chronic stress, we hypothesized that animals experiencing higher levels of social subordination would exhibit a change in mPFC glutamate similar to humans with high PSS scores. As in our prior human work, all monkeys were scanned using single-voxel MR spectroscopy at 3 T focused on change in glutamate levels in medial prefrontal cortex following exposure to an acute stress manipulation. The effectiveness of our acute stress manipulation was assessed using plasma levels of cortisol and the stress-reactive cytokine interleukin-6 (IL-6). As previous research has suggested that submissive behaviors are the best behavioral index for biological adaptations associated with social subordination [40, 48], we tested the extent to which varying levels of submissive behaviors were associated with percent change in mPFC glutamate (%ΔGlu) following stress-induction relative to a control condition. We hypothesized that submissive behaviors related to social subordination would exhibit a negative association with %ΔGlu in mPFC similar to the relationship observed between %ΔGlu in healthy humans following acute stress and the Perceived Stress Scale [28]. Additional variables related to aggressive, affiliative, and anxiety-like behaviors and their associations with (%ΔGlu) and cortisol, interleukin-6 (IL-6), and peripheral measures of systemic inflammation (C-reactive protein; CRP) were also examined.

## Methods

### Animals

Subjects in the current study included 17 adult female rhesus monkeys (*Macaca mulatta*; mean age = 17.9 + /−3.8 years) housed in five social groups of three to five female monkeys each. The social rank of animals within each group was established by the outcome of dyadic agonistic interactions in which subordinate animals emit an unequivocal submissive behavior towards other animals in their groups [34, 35]. Our sample consisted of six high ranking females (most dominant), four middle ranking females, and seven low ranking females (most subordinate). The dyadic observations used to generate rank were collected independently from the behavioral observations collected during the 10-week period prior to the scan (described below). Social groups had been established for at least four years as described previously [49] and were studied in the current study during the fall and winter breeding season (October 2022 -January 2023) [50]. Animals were housed in indoor-outdoor runs (3.7 × 3.7 × 3.7 m) at the Emory National Primate Research Center (ENPRC) Field Station in Lawrenceville, Georgia. Animals were fed a commercially available Purina monkey chow diet (5038) *ad libitum* and had continuous access to water. Seasonal fruits and vegetables were provided daily as a nutritional supplement.

### Ethics statement

The Emory University Institutional Animal Care and Use Committee approved all procedures (IACUC #201700750) in accordance with the Animal Welfare Act and the U.S. Department of Health and Human Services “Guide for Care and Use of Laboratory Animals.” All methods were performed in accordance with the relevant guidelines and regulations.

### Behavioral observations

Behavioral observations were collected to capture rates of aggression, submission, affiliation, and anxiety-like behavior for each group using an established monkey ethogram [49]. Ten, 30-min observations for each group were conducted weekly prior to initiation of the neuroimaging protocol (see experimental design below). These behavioral observations were conducted in the afternoon to create index scores based on counts of individual behaviors per 30 min. Inter-observer reliability was greater than 92%. Aggression directed towards others and received was measured by threats, slaps, grabs, and bites, and submissive behavior was characterized by withdrawals, grimaces, and screams [49]. Affiliative behavior was comprised of engagement in proximity and grooming [49]. Anxiety-like behavior consisted of pacing, body shakes, yawns, and self-scratching [51]. Data were recorded during behavioral observations using a Windows Laptop and the “Hand Obs” program developed by the Center for Behavioral Neuroscience [52].

### Experimental design

**Fig. 1:**
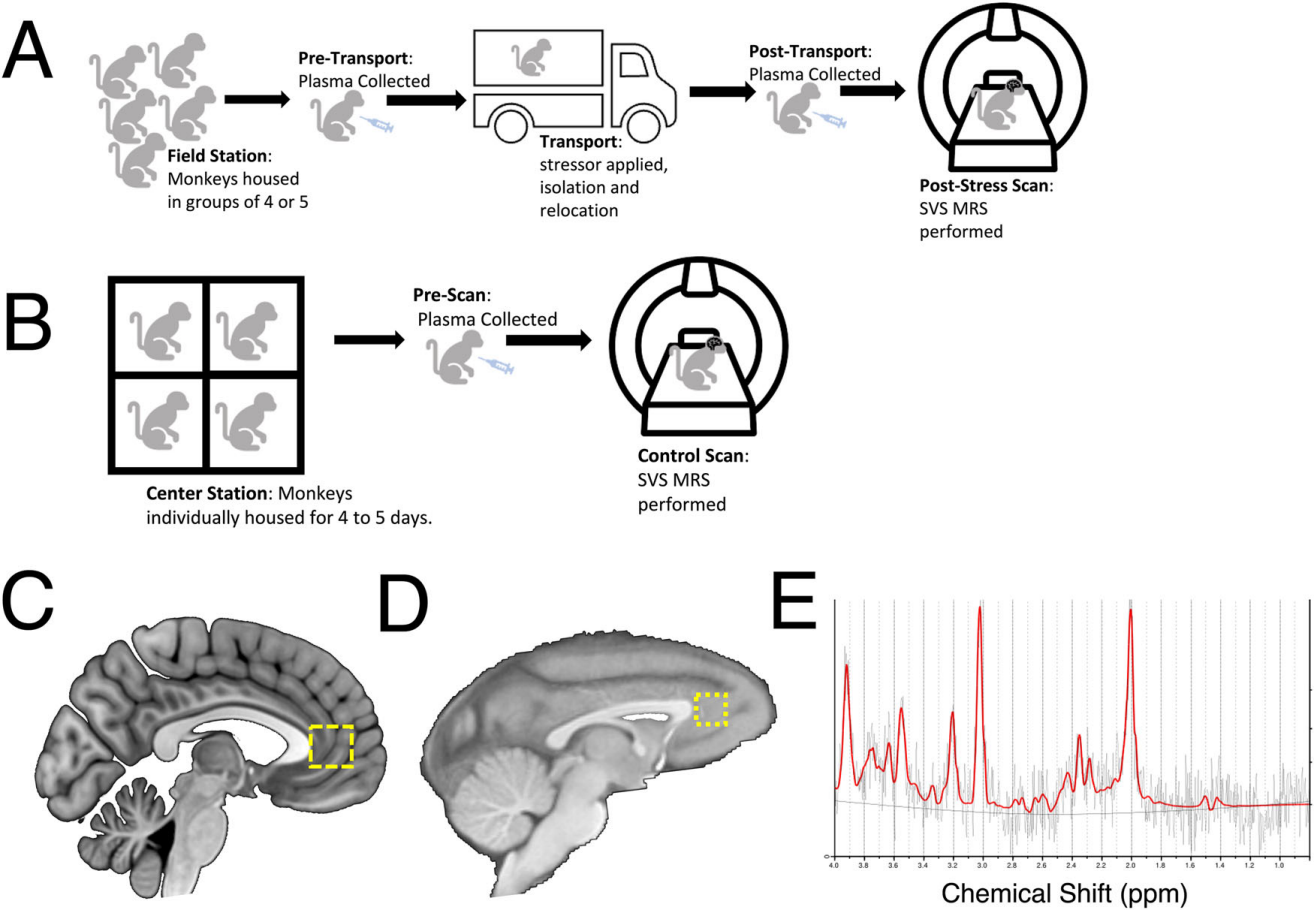
Study design. **A** Schematic diagram of procedures prior to the acute stress scan. **B** Schematic diagram of procedures prior to the control scan. **C** Illustration of representative human voxel placement (from previously published data [28]). **D** Illustration of representative monkey voxel placement. **E** Representative MRS spectra from monkey sample. Grey line reflects raw spectra; red line reflects LC model fit.

Because temporary removal of a female rhesus monkey from her social group to an unfamiliar location is a potent acute stressor [53], animals were removed from their social groups at the ENPRC Field Station and transported to ENPRC main campus wherein they received a MRS scan immediately upon arriving, approximately 40–50 min after leaving the Field Station (Fig. 1A). The animals were recovered from isoflurane anesthesia (see MRS data acquisition methods below) and single housed at the main center for 4–5 five days to acclimate to the new location and return closer to baseline physiological conditions (non-acute stress), after which they received a second “Control” MRS scan (Fig. 1B). Across the animals in the current sample, the Control scan was an average of 4.7 days following the Stress Day scan. We were not able to counterbalance the order of the control and stress scans in the current study due to the lack of an MRI scanner at the ENPRC Field Station.

### Blood sampling and analysis

Blood samples were collected from the saphenous vein at three different times for the assessment of cortisol and IL-6: Blood sample collection occurred:1) immediately following removal from social groups at the field station and prior to transportation stress, 2) upon arrival at the main center after transportation stress and before the first MRS scan, and 3) prior to the Control MRS scan. Samples were assayed for cortisol, IL-6, and CRP (see Supplemental Methods).

### MRS data acquisition and analysis

All in vivo proton magnetic resonance spectroscopy (MRS) experiments were conducted on a Siemens TIM Trio 3 T scanner (Siemens Medical Solutions USA, Malvern, PA) with a Siemens’ receive-only single-loop surface coil (ID = 11 cm) located at the ENPRC Main Center in Atlanta, GA. Animals were sedated prior to intubation and MRS scanning using 5 mg/kg intramuscular dose of Telazol. During MRS scanning, spontaneously breathing animals were intubated and anesthetized using 1–1.5% isoflurane and their heads immobilized in a sphinx position with the home-made head holder. The physiological parameters such as End-tidal CO_2_, O_2_ saturation, blood pressure, heart rate, respiration rate, and body temperature were monitored continuously and maintained in normal ranges [54] following standard veterinary practices [45]. The single voxel MRS was acquired using a conventional point resolved spectroscopy (PRESS) sequence using the parameters: TR/TE = 2000/30 ms, 1024 averages, flip angle = 70°, bandwidth = 1000 Hz, and a 6 × 6 × 6 mm^3^ voxel placed in medial prefrontal cortex (mPFC) so as to approximate the location of our prior human studies (Fig. 1C, D). Structural T1-weighted images were acquired using the 3D magnetization-prepared rapid acquisition with gradient echo (MPRage) sequence to identify the voxel location. Also, an automatic 3D shimming procedure was used to improve the B0 field homogeneity of the whole macaque brain before collecting T1-weighted images [55]. For the second scan, the monkey was immobilized at the same position as the first scan, using the same head holder. High resolution T1 and T2 weighted images were collected using the same protocols and used to define the ROI and to ensure the voxel was placed in the same brain region in the first scan. A manual shimming was conducted before each MRS acquisition. Concentrations of metabolites, including N-acetyl-L-aspartic acid (NAA), creatine and phosphocreatine (total Cr, Cr + PCr, also referred to as “tCr”), total choline (total Cho, primarily phosphocholine + glycophosphocholine, also referred to as “tCho”), Glutamate (Glu), Glx (Glu + Glutamine) were derived from the spectra using the LC Model software concentration (www.s-provencher.com) with the unsuppressed water peak as reference (Fig. 1E).

### Analytic strategy

Based on our prior work, we used submissive behaviors as the best observational metric for characterizing social subordination [40, 48]. Submissive behavior asymmetry was defined as sum of observed submissions given – the sum of observed submissions received over the 10-week evaluation period. Using this metric, individuals with high scores indicated greater social subordination (giving more submissive behaviors than receiving them). We additionally examined associations with aggressive behavior asymmetry, affiliative behaviors and anxiety-like behaviors. Aggressive behavior asymmetry was defined as the sum of observed aggression actions received – aggression actions given. Affiliative behaviors were defined as time spent in proximity with others + time spent receiving grooming + time spent grooming others. Finally, for anxiety-like behavior we considered a single variable representing the total frequency of anxiety-like behaviors described above. All regression analyses were tested with and without the inclusion of age and Control scan glutamate levels as covariates.

Because many of these behavioral measures exhibit a moderate to high degree of collinearity (See Supplemental Materials Figure S1), we also evaluated their association with %ΔGlu using a cross-validated leave-one-subject-out (LOSO) multivariate analysis using partial least squares (PLS) regression [56]. PLS is well-suited to this problem as it is robust to collinearity among predictors. We conducted two PLS models, one using all four variables related to submission and aggression, and one using all three variables related to affiliative behavior. Permutation tests run with 10,000 iterations were used for statistical inference of cross-validated correlations.

### Statistical analysis

To assess changes in plasma cortisol and IL-6, values were log-transformed and a repeated measures ANOVA was used with time as a within-subject factor and a test for a quadratic interaction indicating an elevation during the stress scan relative to values collected at the field station or prior to the Control scan. Follow-up contrasts were performed using paired t-tests. To assess the association between behavioral variables of interest and stress-induced change in glutamate, we first calculated percent change in glutamate (%ΔGlu) using Eq. (1):

1 $$\% \Delta {\rm{Glu}}=\frac{{{\rm{Glu}}/{\rm{Cr}}}_{{\rm{Stress}}\; {\rm{Day}}}-{{\rm{Glu}}/{\rm{Cr}}}_{{\rm{Control}}}}{{{\rm{Glu}}/{\rm{Cr}}}_{{\rm{Control}}}}* 100$$

Associations between behavioral variables and %ΔGlu were tested using ordinary least squares regression. Correction for multiple comparisons was achieved using the False-Discovery Rate [57] and applied to all reported *p*-values in the main text.

## Results

### Associations between social rank and observed behaviors

**Fig. 2:**
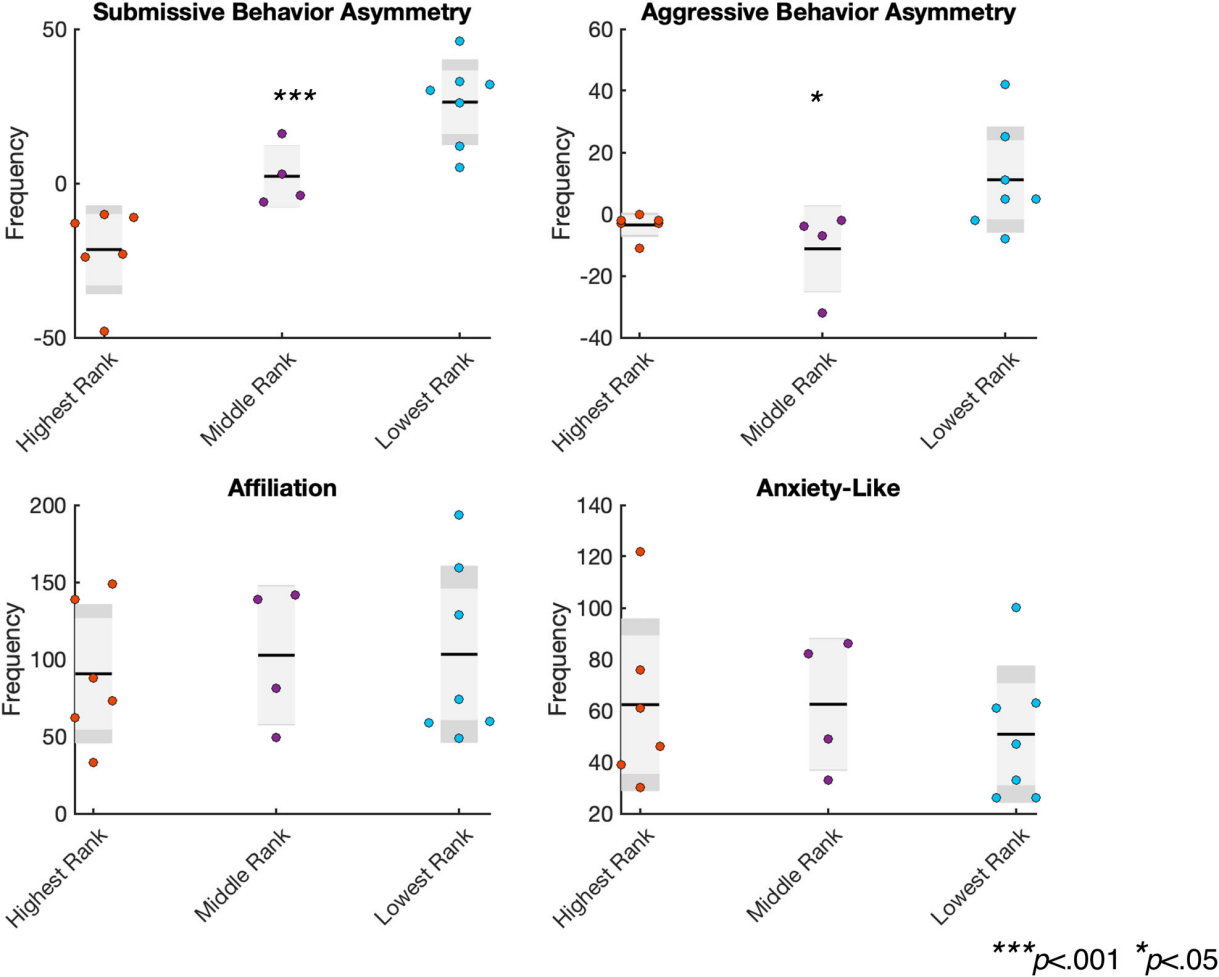
Associations of between social rank as established by dyadic observations and observed behavioral variables collected 10 weeks prior to scanning.

An analysis of the relationship between social rank as established by dyadic observations and observed behavioral variables collected 10 weeks prior to scanning (Fig. 2) revealed a strong effect of social rank on Submission Asymmetry (F_(1,14)_ = 21.03, *p* < 0.000006, η^2^ = 0.75 p_FDR_ = 0.0001), a modest effect of Aggression Asymmetry (F_(1,14)_ = 4.17, *p* < 0.039, η^2^ = 0.37, p_FDR_ = 0.049), and no association with either affiliative behaviors (F_(1,14)_ = 0.12, *p* < 0.891, η^2^ = 0.02) or anxiety-like behavior (F_(1,14)_ = 0.33, *p* < 0.728, η^2^ = 0.04). These data suggest that during the 10-weeks prior to the scan, submissive asymmetry was most strongly associated with established social rank (Fig. 2).

### Effect of stress manipulation on peripheral markers and MRS metabolites

**Fig. 3:**
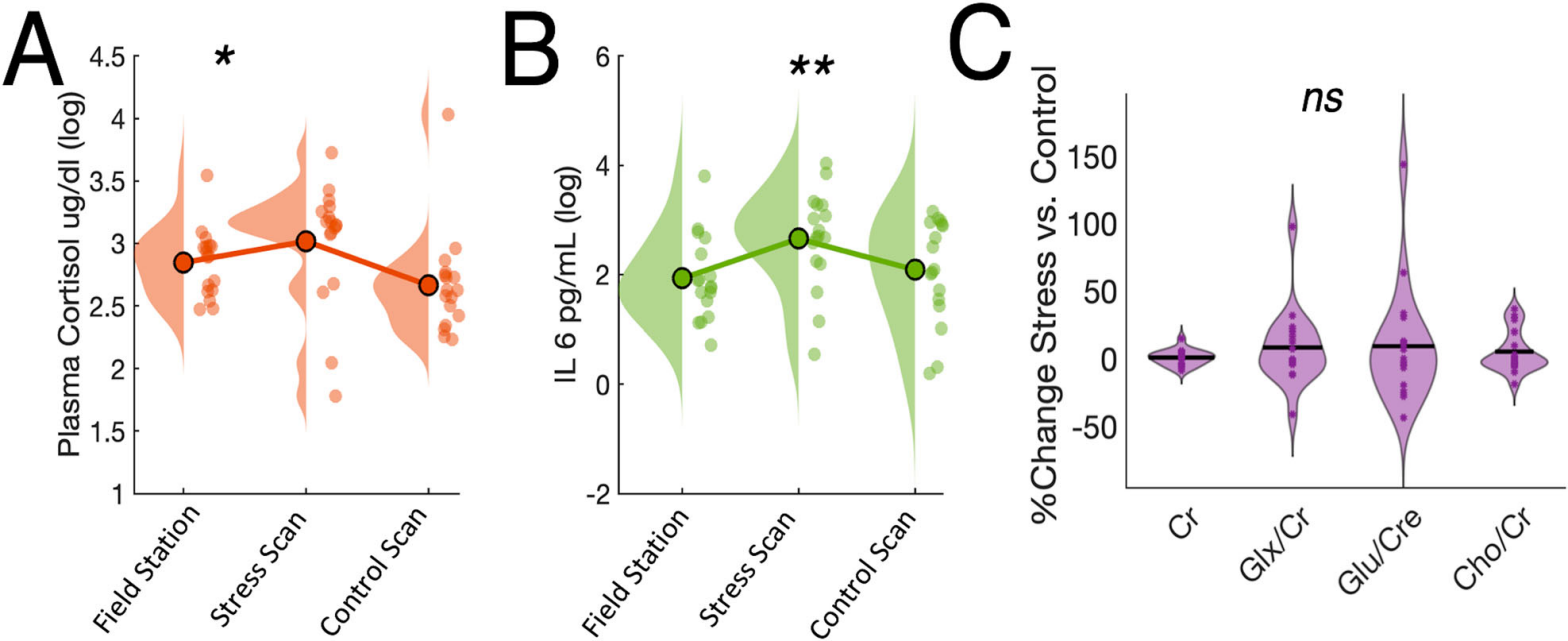
Effects of acute stress procedure on plasma cortisol, plasma IL-6 and MRS metabolites. **A** Effects of acute stress procedure on plasma cortisol showing a significant quadratic interaction. **B** Effects of acute stress procedure on plasma IL-6 showing a significant quadratic interaction. **C** Effects of acute stress procedure on MRS metabolites. No metabolite showed a % change significantly different from zero. *p < 0.05, **p < 0.01.

To verify that our stress-induction procedure was successful, we examined plasma cortisol and IL-6 concentrations. As expected, we observed a significant quadratic effect such that cortisol was elevated immediately prior to the stress MRS scan relative to either the control MRS scan or the sample collected before the stress induction procedure (F_(1,16)_ = 5.10, *p* = 0.038, p_FDR_ = 0.054, η = 0.242) (Fig. 3A). Additionally, there was a significant difference in plasma cortisol immediately prior to the stress scan as compared to the control scan (t_(16)_ = 2.885, *p* = 0.011, p_FDR_ = 0.039, *d* = 0.700). For concentrations of plasma IL-6, a similar quadratic interaction was identified (F_(1,16)_ = 8.59, *p* = 0.010, p_FDR_ = 0.048, η = 0.349) (Fig. 3B), with significant higher values prior to the stress scan relative to the control scan (t_(16)_ = 2.13, *p* = 0.049, p_FDR_ = 0.058, *d* = 0.516). Taken together, both stress-reactive IL-6 and cortisol concentrations confirmed the presence of a stress response immediately prior to the stress scan that returned to baseline before the Control scan was obtained.

In a final analysis examining the main effects of our stress manipulation, we evaluated whether there was a significant increase in MRS metabolites of interest (Glu and Glx) as well as control metabolites (Cr and Cho). Consistent with our prior human work [28], there were no main effects of acute stress vs Control scans for any metabolite tested (Fig. 3C; all p’s > 0.184).

### Univariate associations between %ΔGlu and submissive behavior asymmetry

**Fig. 4:**
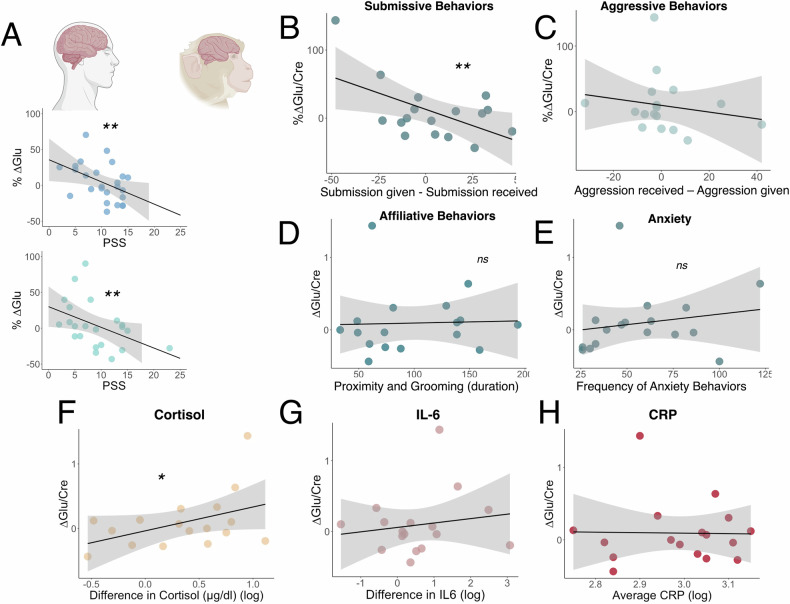
Association between %Δ Glu and measures of recent chronic stress in humans and rhesus macaques. **A** Reproduction of previously published data [28] from two human samples showing the association between perceived stress (PSS scores) and percent change in %Δ Glu. **B**–**H** Data from Rhesus Macaque showing the association between **B** %Δ Glu and submissions given – submissions received. **C** %Δ Glu and aggression received – aggression given. **D** %Δ Glu and affiliative behaviors. **E** %Δ Glu and anxiety-like behaviors. **F** %Δ Glu and stress-induced change in log-transformed plasma cortisol. **G** %Δ Glu and stress-induced change in log-transformed IL-6, and **H** %Δ Glu and basal log-transformed CRP concentrations from plasma collected at the Field Station. **p < *0.05*. *p < *0.10*.

To examine the potential effect of social subordination on mPFC glutamate responses to acute stress, we tested the association between submissive behavior asymmetry and %ΔGlu following acute stress exposure (see Supplemental Materials Table S1 for full metabolite results). Consistent with the pattern observed in humans using the PSS [28] (Fig. 4A), we found that submissive behavior asymmetry was a significant predictor of %ΔGlu (ß = −0.54, *p* = 0.023, p_FDR_ = 0.047) (Fig. 4B), such that animals that gave more submissive behaviors (indicating less social subordination and associated stress), exhibited a higher %ΔGlu, which was attenuated as animals gave more submissive behaviors than they received (indicating greater social subordination and associated stress). Due to the presence of a potential high-influence data-point, this analysis was repeated using a log-transform of %ΔGlu and remined significant (ß = −0.49 *p* = 0.032, p_FDR_ = 0.057). This effect also remained significant when age was included (ß = −0.61, *p* = 0.014, p_FDR_ = 0.040), and remained trend-level significant when age and Control scan glutamate/creatine were included in the model (ß = −0.35, *p* = 0.083, p_FDR_ = 0.084).

### Univariate associations between %ΔGlu and other behavioral dimensions

Next, we examined whether aggressive behavior asymmetry, affiliative behaviors, or anxiety-like behaviors were associated with %ΔGlu following acute stress exposure. No significant effects were observed when examining aggressive behaviors asymmetry (ß = −0.18, *p* = 0.481) (Fig. 4C), affiliative behaviors (ß = 0.03, *p* = 0.896) (Fig. 4D) or anxiety-like behavior ((ß = 0.186, *p* = 0.474) (Fig. 4E). Inclusion of age and Control scan glutamate/creatine did not alter these results. Similar results were obtained when using %ΔGlx or normalization to water instead of creatine (See Supplemental Materials).

### Univariate associations between %ΔGlu and peripheral stress and inflammatory markers

We also tested whether there was any association between %ΔGlu and peripheral measures of the stress response and inflammation. Interestingly, we detected a trend-level association between %ΔGlu and change in log plasma cortisol (ß = 0.44, *p* = 0.077, p_FDR_ = 0.084) that reached significance when controlling for age and Control scan glutamate/creatine in the model (ß = 0.380, *p* = 0.034, p_FDR_ = 0.054) (Fig. 3F). For change in log IL-6 however, there was no evidence of an association (ß = −0.045, *p* = 0.863) (Fig. 3G) and this result was unaffected by inclusion of age and Control scan glutamate/creatine. Lastly, we tested to see whether %ΔGlu was associated with CRP. Neither %ΔGlu (ß = −0.02, *p* = 0.943) nor Control scan Glu (ß = 0.29, *p* = 0.258) were associated with CRP (Fig. 3H). Similar results were obtained when using %ΔGlx or normalization to water instead of creatine (See Supplemental Materials).

### Multivariate associations between %ΔGlu and dimensions of behavior

Similar results were obtained using a cross-validated leave-one-subject-out approach with PLS regression. The four behavioral variables related to submission and aggression asymmetry were significantly predictive of %ΔGlu (cross-validated r = 0.49, permuted *p* = 0.017, p_FDR_ = 0.040), with no association observed of the three behavioral indices for affiliative behaviors (cross-validated r = −0.53, permuted *p* = 0.968). Using a permutation test of cross-validated r contrasts, we found the predictive power of the submission/aggression variables was significantly greater than that for affiliative behaviors (permuted *p* = 0.009, p_FDR_ = 0.064). Similar results were obtained when using to water instead of creatine, but we did not see a significant association with submission and aggression variables and %ΔGlx (See Supplemental Materials).

## Discussion

In the current study, we successfully demonstrated the existence of an adaptive stress response (“AGR”) in a translational non-human primate model. As hypothesized based on prior human and rodent work [24, 26, 28, 58], adult female rhesus monkeys who emit more submissive behaviors than they receive (submissive behavior asymmetry related to lower social rank) showed an attenuated glutamate response in the medial prefrontal cortex to an acute stress challenge. This result makes two significant contributions. First, it isolates agonism-related behavioral variables related to social subordination that appear to drive the AGR in primates. Notably, social subordination has been shown to exert effects on other measures of stress biology, including metabolism, glucocorticoid negative feedback, and gene expression profiles [36, 40–47]. The current results extend these prior peripheral findings to reveal a comparable social-subordination adaptation that occurs within medial prefrontal cortex. Additionally, unlike prior studies identifying trait-like alterations in biological function as a consequence of social subordination, the current results highlight effects on a biological response elicited by an acute stressor, thereby shedding light on the brain systems that may mediate interactions with novel acute stressors in the context of social subordination.

These data show a strong similarity to our previously published work in humans [28], which served as the key motivator for the current study. A limitation of that prior study was its reliance on a subjective assessment of “perceived stress”, which does not take into account any objective threat or deprivation that an individual may be experiencing. In the current NHP sample, we can confidently exclude the possibility that deprivation of basic resources played a meaningful role in the shaping the glutamatergic response, as all animals, regardless of social rank, had equal access to food, water and safety from predators. Additionally, submissive behaviors–but not aggressive behaviors–predicted change in glutamate levels, suggesting that the AGR was driven by the threat of physical aggression rather than actual aggression itself. Our finding that submissive and not aggressive behaviors relate to the AGR is in line with previous findings indicating that submissive behaviors are the best behavioral index for biological adaptations associated with social subordination [40, 48]. As such, social experiences related to subordination within the social hierarchy are sufficient to drive the AGR.

A second contribution of the current work is that it provides a translational NHP model that can now be explored at a mechanistic level. A key limitation of human MRS work is the fundamental ambiguity of the MRS glutamate signal, which reflects glutamate sources from all brain compartments, including neurons, astrocytes, extracellular space, synaptic vesicles and cytosol within neurons and astrocytes (“total glutamate”) [59]. Glutamate plays several critical roles as an excitatory neurotransmitter and an amino-acid contributing to cellular metabolism (e.g., through the TCA-cycle) [60–62]. Prior rodent studies have found positive associations between synaptic glutamate release and the MRS glutamate signal using two-photon imaging [63], and a recent meta-analysis of task-induced changes in glutamate supported the general notion that glutamate signal increases when brain areas are activated by task demands [62]. However, it remains unclear whether this glutamate release is neuronal or glial in origin. Additionally, one human study comparing standard ^1^H proton MRS imaging (the same method used by our group) with ^13^C Carbon MRS that permits estimation of glutamate transmission and neuroenergetics [64, 65], found that the total glutamate was positively correlated with *both* synaptic and metabolic glutamate levels [61], again highlighting the fundamental ambiguity of the ^1^H MRS signal. Indeed, the multiple functional roles of glutamate may contribute to the inconsistent results of clinical ^1^H MRS glutamate studies. Consequently, successful translation of the human AGR into a non-human primate model provides a foundation for understanding the cellular origins and functional roles of glutamate that give rise to the AGR.

While stress-induced reductions in neuronal excitability have been observed in rodents for at least several decades [16] they have been interpreted as both a marker of damage [66–68], as well as a mechanism of adaption [27]. In our prior human work, we observed that multiple samples of healthy controls with no history of psychiatric disease exhibited a robust AGR, while patients diagnosed with current depression did not [28]. Similarly, in the current study we observed a clear AGR as a function of the submissive behavior asymmetry in healthy animals that did not exhibit a behavioral phenotype of depression. Moreover, this effect of social subordination on the AGR was not observed for other dimensions of psychopathology-related phenotypes, such as affiliative and anxiety-like behavior. While we have previously found subordination in female macaques is associated with lower rates of affiliation with others and greater rates of anxiety-like behavior [38, 39], other studies have shown no such relationships and highlight that group composition, seasonality, and other contextual factors are important for social status effects on affiliative and anxiety-like behavior in NHPs [40, 69, 70]. The lack of a relationship between these other socioemotional behaviors and the AGR in the current study suggests that submissive behavior asymmetry, a direct measure of social subordination in macaques, is a potent psychosocial factor that impacts stress physiology [48]. Interestingly, submissive behavior was also most strongly associated with social rank. Finally, the effect of social subordination on the AGR remained after controlling for Control scan Glu levels, suggesting the association was specific to the acute stress manipulation. Overall, these data would support the interpretation of the AGR as a marker for a normal physiologic function that presumably supports allosteric regulation of stress exposure.

There are several other notable advantages of the current back-translation approach to NHPs. In highly socialized species such as humans and NHPs, the links between stress and adverse behavioral outcomes are greatest for circumstances in which the stressor involves social subordination, rejection, or isolation [30–32]. Moreover, the social networks and prefrontal brain morphology of rodents may limit their relevance to human stress-borne disease. The current findings of dysfunction of glutamatergic system extend previous findings describing alterations in dopaminergic [43, 44], serotonergic [45, 46], and GABAergic systems [47] in prefrontal regions critical for emotion regulation in socially subordinate monkeys.

Finally, we examined associations between stress-evoked glutamate and C-reactive protein and IL-6, measures of systemic inflammation and stress-reactive inflammatory responses, respectively. Inflammation has been widely associated with acute and chronic stress exposure [71] and risk for stress-linked disorders such as depression [72–74]. Moreover, inflammation has been associated with alterations in MRS Glutamate [75–77]. Here, we did not detect any clear associations between either systemic inflammation (CRP) or stress-induced inflammation (IL-6) and stress-related changes in mPFC glutamate (Fig. 3F, G). Further research will be required to determine whether inflammation plays a role in the AGR or its dysfunction in the context of psychiatric disorders.

### Limitations

While the current work has many strengths, there are several important limitations. First, our results focused on stress-related adaptation of glutamate, but not GABA. Preclinical studies have found that modulation of GABAergic interneurons may be key drivers of stress adaptation [78, 79], and the observed association between submission behavior and glutamate may partially reflect the impact of interneuron plasticity on total glutamate levels. Due to technical limitations of our MRS sequence, we were not able to image both glutamate and GABA reliably, and prioritized glutamate given our prior human work. That said, future studies should investigate the role of GABA modulation in adaptation to stress.

A second limitation was that our sample is small for individual differences analyses, even though it is a relatively large sample in the context of NHP neuroimaging studies. Indeed, some of our results became only marginally significant after correcting for multiple comparisons using FDR correction, which was likely due in part to our smaller sample size. We sought to address this power by performing both univariate and multivariate analyses, which have different limitations in the context of low samples. An additional limitation is that animals were housed in isolation prior to our Control scan. This was necessary, as there are no MR scanning facilities at the field station. Moreover, our use of transportation from the field station as an acute stressor meant that we were unable to counterbalance the order of scans, and all animals received the stress scan first. Importantly, we did confirm that both IL6 and plasma cortisol showed a specific increase during the acute stress scan that returned to basal levels prior to the Control scan (Fig. 2A, B). Nevertheless, it is possible the Control scan does not reflect a true return to baseline conditions.

Another limitation was that our sample was comprised only of females, thereby precluding our ability to generalize our findings to males. Although the focus on females was necessary due to the fact that adult male rhesus monkeys cannot be housed in similar same-sex social groups at the ENPRC, our findings in females are important as women are at greater risk than men for stress-related psychopathology [80–83]. An additional limitation is that animals received sedation and anesthesia prior to scanning, which could alter glutamate measurements. While sedative and isoflurane doses were consistent across the two scans, we cannot rule out the possibility of an interaction between these agents and stress in relationship to MRS measures of glutamate. A final limitation is that the signal-to-noise ratio (SNR) was low in our sample, resulting in a group-average Cramer-Rao Standard Deviation that was > 20. This may have contributed to the absence of a main effect of acute stress on metabolite levels. That said, our prior human studies with very good SNR (Glutamate Cramer-Rao Standard Deviations < 10) also did not show a main effect of acute stress across all participants; indeed, only individuals with low levels of chronic stress showed an increase in Glu following acute stress [28]. This is consistent with what was observed for monkeys with less submission asymmetry. While the current sample was too small to perform sub-group analyses, these results are generally consistent with the pattern of our prior human work.

## Conclusion

In the current study we sought to test the generalizability of a previously observed AGR in humans to NHPs. Consistent with our prior work in humans who reported varying levels of chronic stress, we found that mPFC glutamate levels in *rhesus macaque* assessed by MRS following an acute stressor were largest for animals with the largest asymmetry in submissive behavior, which was associated with higher ranking animals. In contrast, mPFC glutamate levels declined as exposure to social subordination increased. This effect was specific to behavioral measures of subordination and was further associated with acute stress-evoked changes in plasma glucocorticoids. Taken together, these data further establish the AGR in the mPFC as a common biomarker adaptation to an acute stressor as a function of chronic stress exposure.

## Supplementary information

Supplemental Material

## Data Availability

Behavioral and MRS data will be provided upon reasonable request.
