# Supplementary material for: Medial prefrontal glutamate response to acute stress is associated with social subordination in female rhesus macaques
Source: Transl Psychiatry. 2025 Mar 29;15:107. doi: 10.1038/s41398-025-03334-2 (PMC11954936; doi:10.1038/s41398-025-03334-2)
Supplement: Supplementary file 1 — Supplemental Material [file 41398_2025_3334_MOESM1_ESM.docx]

SUPPLEMENTAL MATERIALS FOR:

Medial prefrontal glutamate response to acute stress is associated with social subordination in female rhesus macaques

Michael T. Treadway^1,2*^ Samantha A. Betters^1^, Jessica A. Cooper^2^, Chun-Xia Li^3^, Xiaodong Zhang^3^, Vasiliki Michopoulos^2,3*^

^1^Department of Psychology, Emory University, Atlanta, GA 30322

^2^Department of Psychiatry and Behavioral Sciences, Emory School of Medicine, Atlanta, GA 30322

^3^Emory National Primate Research Center, Atlanta, GA 30322

**SUPPLEMENTAL METHODS**

*Biomarker Analyses:* All blood samples collected were assayed using commercially prepared immunoassay kits for cortisol (Arbor Assay, K003-H1) and IL-6 (Abcam Monkey IL-6, ab242233) in the Biomarker Core Laboratory at the ENPRC. The cortisol assay had an inter- and intra-assay coefficient of variation of 9.58% and 6.22%, respectively. The IL-6 assay had an inter- and intra-assay coefficient of variation of 11.9% and 11.7%, respectively. One aliquot of plasma collected immediately following removal of animals from their social groups and prior to transportation stress was assayed for CRP (ALPCO, KR9710s). The CRP assay had an intra-assay coefficient of variation of 2.03%.

**SUPPLEMENTAL RESULTS**

Figure S1: Zero-order Correlations Among Behavioral Variables.


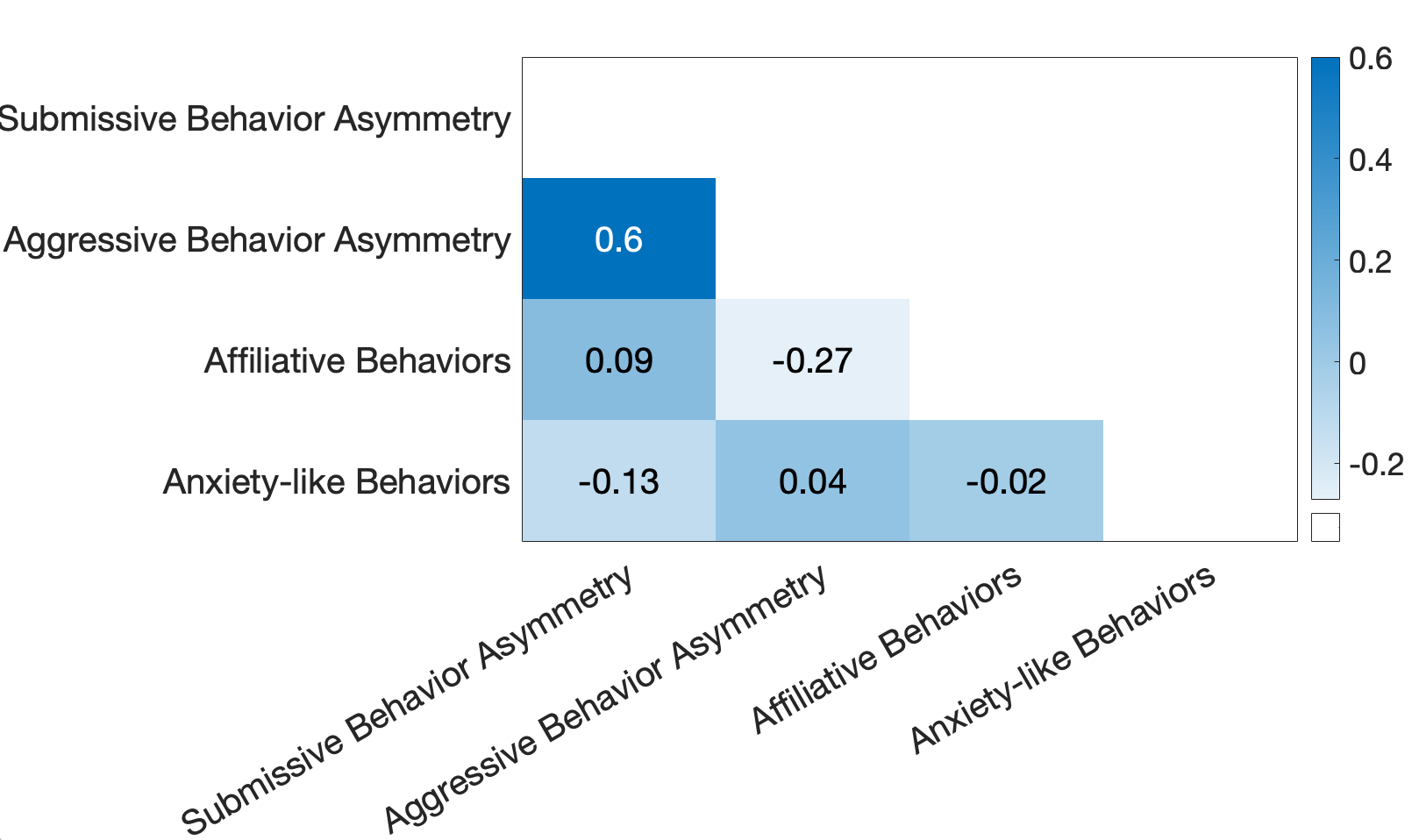

Table S1. MRS METABOLITE VALUES AND CRAMER RAO LOWER BOUNDS (CRLB)

true *r* = Leave-one-subject-out (LOSO) cross-validated Pearson correlation between training and test data.

permuted *r* = Leave-one-subject-out (LOSO) cross-validated Pearson correlation between permuted training and test data.

true MSE = Leave-one-subject-out (LOSO) cross-validated mean squared error between training and test data.

permuted MSE = Leave-one-subject-out (LOSO) cross-validated mean squared error between permuted training and test data.
